# Antioxidative activity of different parts of the plant *Lepidium sativum* Linn

**DOI:** 10.1016/j.btre.2014.05.006

**Published:** 2014-06-04

**Authors:** Jency Malar, K. Chairman, Anita R.J. Singh, J. Shifa Vanmathi, A. Balasubramanian, K. Vasanthi

**Affiliations:** a Department of Biotechnology, Sri Paramakalyani College, Alwarkurichi, Tirunelveli 627412, Tamilnadu, India; b Department of Zoology, Sri Paramakalyani College, Alwarkurichi, Tirunelveli 627412, Tamilnadu, India; c Women's Christian College, Autonomous Institution University of Madras, Chennai, India^1^; d Sarah Tucker College, Palayamkottai, Tirunelveli, India; e Department of Zoology, Sri Parasakthi College for Women, Courtallam, Tirunelveli, Tamilnadu, India

**Keywords:** *Lepidium sativum*, Antioxidative activity, DPPH, Ascorbic acid, Glutathione, Fe^3+^–Fe^2+^ Transformation Ability

## Abstract

*Lepidium sativum* Linn. has been used in traditional and folklore medicine for the treatment of bronchial asthma, diabetes, local and rheumatic pain. An ethanolic extract of cress (*L. sativum* L.) shoot, leaf, stem and seed has been studied for antioxidative active against 1,1-diphenyl-2-picrylhydrazyl (DPPH), total glutathione S-transferase assay, reduced glutathione activity, reducing power (Fe^3+^–Fe^2+^ Transformation Ability), and ascorbic acid is also estimated. The percentage yields of free radical scavenging activity (DPPH) obtained for different ethanolic extracts of *L. sativum*. Supreme scavenging activity was detected in shoot (12.19 ± 02%) and least in stem (2.69 ± 05%). The activity of total glutathione S-transferase enzyme was found to be more in seed (9600 ± 56.3 μg/ml) than other plant parts. The reduced glutathione content of the ethanolic extracts of *L. sativum* was found to be more in leaf (9 ± 0.2 μg/ml). In the reducing power assay, ethanolic extracts gives the optical density in increasing concentration in all plant parts it shows that it has the reducing ability of Fe^3+^–Fe^2+^. Presence of vitamin C was tested. It was found that the shoot extract has highest amount of vitamin C. The results of present data were shown that the ethanolic extract of *L. sativum* L. plant parts have contributed high potential in vitro antioxidant activity.

## 1 Introduction

Higher plants are the main source of medicine throughout the human history. A multitude of plant species are still widely used for the traditional as well as modern systems of medicine. As per the statistic of WHO, up to 70% of population living in developing countries depend on plants for primary health care and 25% of the prescriptions in modern medicine got ingredients of plant origin. Most of the discussion and debates on medicinal plants relate to those species distributed in the tropical countries such as India, China, Malaysia, and Brazil. It is also known that most of the expensive lifesaving drugs manufactured by Western pharmaceutical companies are from medicinal and aromatic plant that are being reported as intact plant or in the form of crud extract from the tropical region. Species of *Lepidium* are an exception as they are mostly of temperate origin and are known to yield live saving drugs. *Lepidium sativum* is the most popular of all *Lepidium* belonging to the family *Cruciferae* grown in India, Europe and US is an underutilized crop. The herb is highly used by the rural and tribal people in curing various disorders. The present project enumerates various traditional and ethno-medicinal utility of the plant [5].

Garden cress (*L. sativum*) is a fast-growing, edible herb that is botanically related to water cress and mustard, sharing their peppery, tangy flavour and aroma. In some regions, garden cress is known as garden pepper cress, pepper grass, pepperwort or poor man's pepper. It is a perennial plant, and an important green vegetable consumed by human beings, most typically as a garnish or as a leaf vegetable. Garden cress is found to contain significant amounts of iron, calcium and folic acid, in addition to vitamins A and C. This annual plant can reach a height of 60 cm (∼24 in.), with many branches on the upper part. The white to pinkish flowers are only 2 mm (1/12 of an inch) across, clustered in branched racemes. The garden cress produces an orange flower suitable for decorative use and also produces fruits which, when immature, are very much like caper berries. Garden cress is also used as a medicine in India in the system of Ayurveda. It is used to prevent post-natal complications; the seeds of this plant perform as an aperient when boiled with milk (Dahanukar et al., 2000).

*L. sativum* has been widely used to treat a number of ailments in traditional system of medicine throughout India. Preliminary phytochemical study of *L. sativum* following standard procedures showed presence of flavonoids, coumarins, sulphur glycosides, triterpenes, sterols and various imidazole alkaloids. The major secondary compounds of this plant are glucosinolates [3]. The alkaloids of *L. sativum* are member of the rare imidazole alkaloids that is known as lepidine. Despite the widespread traditional/edible uses of *L. sativum*, there is very few pharmacological works done. Phytopharmacological screening of alkaloid and glucosinolates are untouched so far [10].

Correct identification and quality assurance of the starting materials is an essential prerequisite to ensure reproducible quality, which will provide safety and efficacy of herbal medicine. This study was undertaken to generate standardized data on various pharmacognostical, phyto and physico-chemical characteristics of the plant materials. The outcome of the present study will be helpful in identification, authentication and quality control of the plant materials.

## 2 Materials and methods

### 2.1 Plant material

The plant *L. sativum* was grown in the laboratory of Women's Christian college, Chennai, Tamilnadu, India. The shoots, leaves, seed and stem were shade dried and pulverized using mortar and pestle separately and stored in a closed vessel for further use.

### 2.2 Preparation of extracts

The powdered parts such as shoot, seed, stem and leaves of *L. sativum* collected from the laboratory, were extracted with ethanol using soxhlet extraction apparatus. The ethanolic extracts were then dried under reduced pressure and controlled temperature. The crude ethanol free dried powdered materials were used for experiments. The extracts were separately dissolved in dimethylsulfoxide (DMSO) and used for specific assays.

### 2.3 Free radical scavenging assay

Ethanolic extracts of *L. sativum* seed, stem, leaf and whole plant were collected. 30 mg of each extract was weighed and dissolved in 3 ml of DMSO solution and mixed well. This extract was used for the determination of DPPH scavenging activity. In the tube labelled as test 1 ml of DPPH solution was mixed with 450 μl tris–HCL solution and 100 μl of extract such as seed, stem, leaf and whole plant was added to the mixture and kept for 10 min at room temperature. To the control tube 100 μl of distilled water was added and incubated. Absorbance of control tube and the sample tubes was measured at 517 nm. Percentage DPPH scavenging activity was calculated using the following formula [11]:

$$\%\;\text{DPPH scavenging activity}=\frac{(\text{control absorbance}-\text{sample absorbance})\times 100}{\text{Control absorbance}}$$

### 2.4 Total glutathione S-transferase assay

Ethanolic extracts of *L. sativum* seed, stem, leaf and whole plant were collected. 30 mg of each extract was weighed and dissolved in 3 ml of DMSO solution and mixed well. This extract was further used. A clean 96-well plate was taken. 150 μl of phosphate buffer and 20 μl of glutathione solution were added to blank and sample wells. 20 μl of phosphate buffer and 20 μl of plant extract were added to blank and sample wells, respectively. Reaction was initiated by adding 10 μl of CDNB to both the wells and mixed well. The absorbance was read at 340 nm up to 5 min with an interval of 1 min using plate reader at 250 °C. Change in absorbance per minute was calculated using the following formula [13]

$$\text{Delta absorbance}\;340\;\text{nm/min}=\frac{A_{340}(\text{time}\;2)-A_{340}(\text{time}\;1)}{\text{time}\;2(\min)-\text{time}\;1(\min)}$$

$$\text{GST activity}\;(\text{nmol/ml/min})=\frac{\text{Delta absorbance}\;340\;\text{nm/min}\times \text{total volume of assay system}\;(0.2\;\text{ml})\times \text{sample dilution}}{0.00503\;\mu {\text{M}}^{-1}\times \text{original volume of enzyme taken for analysis}\;(0.02\;\text{ml})}$$

0.00503 μM^−1^ = extinction coefficient of CDNB at 340 nm. The actual extinction coefficient for CDNB is 0.0096 μM^−1^ cm^−1^. The value has been adjusted to the path lengths of the solution in the well.

### 2.5 Reduced glutathione assay

Ethanolic extracts of *L. sativum* seed, stem, leaf and whole plant were collected. 30 mg of extract was weighed and dissolved in 3 ml of DMSO solution and mixed well. This extract was used further. 100–400 μl of glutathione standard solution was pipetted in different test tubes and the final volume was made up to 1 ml. 3 ml of phosphate buffer was added and mixed well. 0.5 ml of DTNB was added to all the tubes and incubated at room temperature for 5 min. Absorbance was taken at 412 nm within 10 min 100 μl of extract was treated as above and the absorbance was taken at 412 nm. Blank tubes having all the reagents except glutathione solution and the extract were also included. Graph was plotted using glutathione concentration in *X*-axis and absorbance at 412 nm in *Y*-axis and the glutathione content in plant extract was found out using standard graph [4].

### 2.6 Determination of reducing power (Fe^3+^–Fe^2+^ Transformation Ability)

Ethanolic extracts of *L. sativum* seed, stem, leaf and whole plant were collected. Various concentrations of the extracts (0, 1, 2, 3, 5, 7, 8, 11) in 1 ml of water were mixed with phosphate buffer (2.5 ml, 0.2 mol, pH 6.6) and1% potassium ferricyanide (2.5 ml). The mixture was incubated at 50 °C for 20 min. Aliquots of trichloroacetic acid (2.5 ml, 10%) were added to the mixture. Centrifuge the mixture at 3000 × *g* for 10 min. Upper later of solution (2.5 ml) was mixed with distilled water (2.5 ml) and freshly prepared ferric chloride solution (0.5 ml, 0.1%). The absorbance was measured at 700 nm [12].

### 2.7 Estimation of ascorbic acid

Pipette out 5 ml of standard ascorbic acid in a conical flask. To this add 10 ml of 4% oxalic acid place in an ice bath and titrate against the dye in a burette. The end point is the appearance of pale pink colour. Repeat the procedure for concordant values. The amount of dye consumed is equal to the amount of ascorbic acid present. Pipette out 5 ml of experimental solution in a conical flask and add 10 ml of 4% oxalic acid. Titrate against the dye till the appearance of pale pink colour.

## 3 Results and discussion

### 3.1 Free radical scavenging activity

The percentage yields of free radical scavenging activity obtained for different ethanolic extracts of *L. sativum* are stem (2.69 ± 05%), leaf (10.21 ± 09%), seed (11.63 ± 03%) and shoot cultures (12.19 ± 02%). For scavenging activity, hydrogen donating ability of the extract to the free radical DPPH was determined. When DPPH is scavenged, the deep violet colour turns to pale yellow which can be determined spectrophotometrically. All extracts showed scavenging activity in concentration dependent pattern [7].

In the ethanolic extract of *L. sativum*, shoots exhibited higher scavenging activity than the seed (Table 1). This might be due to the higher content of the total polyphenolic compounds in the seed. Leaf extract exhibited higher scavenging activity and the stem extract showed the lowest scavenging activity among all the extracts.

**Table 1:** Estimation of free radial scavenging activity with the ethanolic of extracts of different parts of *Lepidium sativum*.

Contents	Concentration (μg/ml)	O.D. (512 nm)	% DPPH scavenging activity
Blank	–	0.705	–
Stem	100	0.686	2.69 ± 0.5
Leaf	100	0.633	10.21 ± 0.7
Whole plant	100	0.619	12.19 ± 0.2
Seed	100	0.623	11.63 ± 0.3

O.D., optical density.

The results compared with the published results of Ho et al. [8] and Choi et al. [1] in different *Lepidium* species. Methanol and chloroform extracts (0.01 mg dw/ml) of *Hypericum cerastoides* significantly quenched DPPH (84.2% ± 0.3), although it demonstrated a low total antioxidant activity (19.5 ± 0.8 μM TE/g). The scavenging ability of *Hypericum perforatum* has significant values 77.6% ± 0.5 or DPPH and corresponds to the presence of high quality of phenolic compounds.

The scavenging activity might be due to the presence of total polyphenolic compounds. These polyphenolic compounds include flavonoids, anthraquinones, anthocyanidins, xanthones and tannins [2]. These compounds have been reported to scavenge free radicals, superoxide and hydroxyl radical by single electron transfer. Although these phytochemicals were not assayed for *L. sativum* in the present study, it is presumed the species is rich in such phenolic compounds [9].

### 3.2 Total glutathione S-transferase activity

The activity of glutathione S-transferase enzyme in the ethanolic extracts of *L. sativum* using glutathione and 1-chloro-2,4-dinitrobenzene was found to be stem 2000 ± 52.6 nmol/ml/min, leaf 8800 ± 76.4 nmol/ml/min, shoot 6000 ± 43 nmol/ml/min and seed 9600 ± 56.3 nmol/ml/min (Table 2).

**Table 2:** Estimation of total glutathione S-transferase activity (content) of the ethanolic extracts of different parts of *Lepidium sativum*.

Contents	Concentration (μg/ml)	O.D. (340 nm)					Total GST content (nmol/ml/min)
		1 min	2 min	3 min	4 min	5 min	
Blank	–	0.038	0.04	0.04	0.044	0.039	–
Stem	100	1.161	1.168	1.149	1.171	1.133	2000 ± 52.6
Leaf	100	1.973	1.997	1.966	1.986	1.955	8800 ± 76.4
Shoot	100	1.920	1.937	1.940	1.936	1.933	6000 ± 43
Seed	100	2.043	2.069	2.042	2.042	2.012	9600 ± 56.3

O.D., optical density; GST, glutathione S-transferase.

These values confirm extracts contain enhanced antioxidant activity. Similar high activity of glutathione S-transferase activity noticed in such other plants such as *Zygophyllacae* and *Euphorbiaceae* has also been related positively to their antioxidant potential (Muhammad Rizwan-ul-Haq et al., 2010).

### 3.3 Reduced glutathione activity

The reduced glutathione content of the ethanolic extracts of *L. sativum* was found to be in stem 8 ± 0.46 μg/ml, leaf 9 ± 0.2 μg/ml, shoot 6 ± 0.31 μg/ml and seed 4 ± 0.12 μg/ml (Table 3). The intracellular reactive oxygen species assay which determines the intracellular levels of glutathione (GSH) reveals release of increased antioxidants in all the extracts of *L. sativum* [14]. The results from the study suggest levels of decreased glutathione with corresponding enhanced antioxidant activity. Among the extracts highest value was observed in leaf 9 μg/ml.

**Table 3:** Estimation of reduced glutathione content in the ethanolic extracts of different parts of *Lepidium sativum*.

Contents	Concentration (μg/ml)	O.D. (512 nm)	Glutathione content (μg/ml)
Blank	–	0.00	–
S1	10	0.50	–
S2	20	1.00	–
S3	30	1.70	–
S4	40	2.50	–
Stem	100	0.40	8 ± 0.46
Leaf	100	0.45	9 ± 0.2
Shoot	100	0.30	6 ± 0.31
Seed	100	0.21	4 ± 0.12

O.D., optical density.

### 3.4 Reducing power (Fe^3+^–Fe^2+^ Transformation Ability)

In the reducing power assay, the presence of antioxidants in the samples would result in the reduction of Fe^3+^–Fe^2+^ by donating an electron. Amount of Fe^2+^ complex can be then monitored by measuring the formation of pearl's Prussian blue at 700 nm indicates an increase in reductive ability [6].

**Fig. 1:**
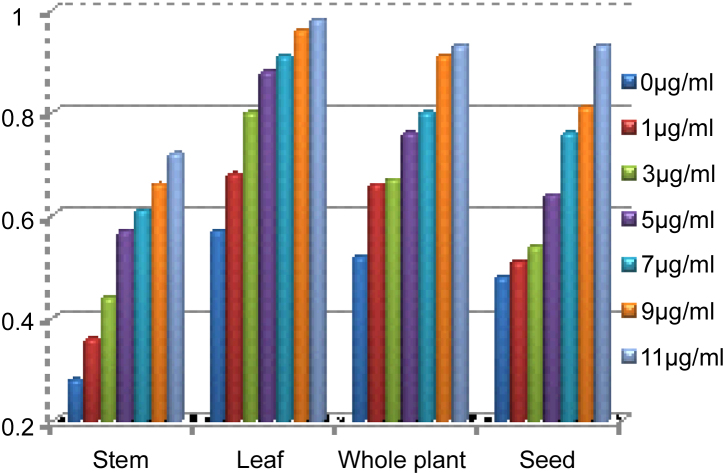
Valuation of reducing power (Fe^3+^–Fe^2+^ Transformation Ability) in the ethanolic extracts of different parts of *Lepidium sativum*.

Ethanolic extracts of *L. sativum* gives the optical density in increasing concentration in all plant parts (Table 4 and Fig. 1) it shows that it has the reducing ability of Fe^3+^–Fe^2+^.

**Table 4:** Estimation of reducing power in the ethanolic extracts of different parts of *Lepidium sativum*.

Concentration of plant extract (μg)	Optical density (700 nm)			
	Stem	Leaf	Shoot	Seed
0	0.28	0.57	0.52	0.48
1	0.36	0.68	0.66	0.51
3	0.44	0.80	0.67	0.54
5	0.57	0.88	0.76	0.64
7	0.61	0.91	0.80	0.76
9	0.66	0.96	0.91	0.81
11	0.72	0.98	0.93	0.93

### 3.5 Estimation of ascorbic acid

The amount of ascorbic acid (vitamin C) was estimated. The whole plant showed 11.74 ± 0.83 mg, and stem showed 11.74 ± 0.83 (Table 5) of ascorbic acid.

**Table 5:** Estimation of ascorbic acid in the ethanolic extracts of different parts of *Lepidium sativum*.

Plant parts	Weight (mg)
Seed	9.68 ± 0.72
Stem	11.74 ± 0.83
Whole plant	12.5 ± 0.60
Leaf	7.4 ± 0.38

## 4 Conclusion

In this work the herbal plant *L. sativum* was selected for the biological studies, which consist of several medicinal benefits for humans. Ethanolic extracts of *L. sativum* was also analyzed for free radical scavenging and antioxidant activities using DPPH assay, glutathione S-transferase activity and quantifying reduced glutathione content. The results suggested that the extracts contain high antioxidant activities and therefore form a potential source of natural antioxidant compounds.
